# Synthesis of Fe_2_O_3_/TiO_2_ Photocatalytic Composites for Methylene Blue Degradation as a Novel Strategy for High-Value Utilisation of Iron Scales

**DOI:** 10.3390/ma17184546

**Published:** 2024-09-16

**Authors:** Li Liu, Zhenghao Cui, Bo Feng, Mengjing Sui, Huaqin Huang, Zhaoyang Wu

**Affiliations:** 1 School of Metallurgical Engineering, Anhui University of Technology, Maanshan 243002, China; 18855579760@163.com (L.L.);; 2 Anhui International Joint Research Center for Metallurgical Processes and Systems Science, Anhui University of Technology, Maanshan 243002, China

**Keywords:** photocatalytic composite material, iron scales, photocatalytic activity, TiO_2_, recycling

## Abstract

In this study, novel Fe_2_O_3_/TiO_2_ photocatalytic composites were synthesised by combining traditional oxidation roasting with the sol-gel method, using low-cost metallurgical waste (iron scales) as the raw material. The characterisation results revealed that the oxidised iron scales could be transformed into high-purity and porous Fe_2_O_3_ particles through oxidation roasting, thereby providing additional sites for the adsorption process and thus serving as an effective carrier for TiO_2_-based photocatalytic materials. During the sol-gel process, TiO_2_ was loaded onto the synthesised Fe_2_O_3_ particles, generating core-shell heterostructure Fe_2_O_3_/TiO_2_ photocatalytic composites. Under visible light irradiation for 90 min, the Fe_2_O_3_/TiO_2_ photocatalytic composites achieved a remarkable methylene blue removal rate (97.71%). This reaction process followed the quasi-first-order kinetic model with a rate constant of 0.038 min^−1^. The results have demonstrated that this combination of various components in the Fe_2_O_3_/TiO_2_ photocatalytic composites improved the adsorption, light utilisation, and charge separation effect of the photocatalysts. Moreover, the material exhibited favourable stability and recyclability, making it a decent candidate for the treatment of wastewater from the biochemical industry. Therefore, this study provides a new strategy for improving the photocatalytic activity of TiO_2_ and expanding the high value-added utilisation of iron scales.

## 1. Introduction

Since the beginning of the 21st century, the global emission of wastewater from the biochemical industry has reached alarming levels, which contain high levels of organic pollutants that are extremely resistant to degradation. Even at low concentrations, these pollutants exhibit considerable chromaticity, reducing light transmission within natural waterbodies. Consequently, this severely affects the photosynthesis process of aquatic plants and impedes the growth of aquatic animals. Additionally, exposure to these organic pollutants and their intermediates poses a long-term risk to human health because they may contain carcinogens, teratogens, and mutagens [1]. The removal of these organic pollutants and dyes still presents a challenge owing to their low chemical oxygen demand and unique biochemical properties, which result in their inadequate removal through conventional treatment methods used for wastewater discharged from the biochemical industry [2,3,4].

Photocatalysis has emerged as a highly promising technology for wastewater treatment because it can completely degrade organic pollutants into small inorganic molecules, such as CO_2_ and H_2_O, without causing any secondary pollution [5]. Among the various photocatalysts, TiO_2_ is preferred for wastewater treatment owing to its exceptional ability to degrade organic dyes. This ability is attributed to its high chemical stability, non-toxicity, and long catalytic lifespan [6,7,8]. Nevertheless, the bandwidth of anatase TiO_2_ is approximately 3.2 eV, restricting its capacity to absorb and harness ultraviolet (UV) light with wavelengths less than 387 nm. This absorption range represents only 4% of the sunlight spectrum, considerably constraining the applicability of anatase TiO_2_ in the visible region. Furthermore, there are several challenges, such as TiO_2_ aggregation, TiO_2_ poisoning and inactivation, and suspended TiO_2_ powder retrieval, associated with reusing TiO_2_ catalysts, increasing the cost of its implementation [9]. Loading TiO_2_ onto carriers is an effective way to overcome these challenges because it prevents the photocatalytic agglomeration of TiO_2_, reduces its dosage, and increases its light response to visible wavelengths. Furthermore, carriers with high specific surface areas and strong adsorption properties can be used for the targeted enrichment of pollutants in water and air, enhancing the efficiency and recyclability of photocatalytic materials [10,11,12,13]. For example, Christoforidis used β-Fe_2_O_3_ as the catalyst carrier, improved the optical properties of composite materials, and obtained composites with narrower bandgap energy and higher visible light absorption efficiency [14]. To date, various carrier materials, mainly including metals (e.g. singlet metals, metal ions, and metal oxides) [15,16,17], glasses (e.g. silica glass fibres, silica glass, and basalt fibres) [18,19,20], adsorbents (e.g. activated carbon, zeolite, and graphite), vermiculite two-dimensional mixed-layer interstratified structures [21], and polymeric organic polymers (e.g., polypropylene, polyimide and polyvinyl alcohol) have been developed [22,23,24]. TiO_2_ can be loaded onto carrier materials using various methods, including powder sintering, coupling agents, the sol-gel method, the hydrothermal method, and chemical vapour deposition. However, industrial applications of these photocatalytic composites are limited by neither high synthetic costs nor poor photocatalytic performance [25,26,27].

Iron scales (ISs) are metallurgical solid waste generated during the manufacturing of continuous casting billets or ingots, heating furnaces, and rolling lines [28]. ISs account for approximately 2%–3% of steel production in steel mills. The global crude steel production in 2022 was 1.885 billion tonnes, generating over 37.7 million tonnes of ISs, which may cause soil and air contamination if they are not properly disposed of [29]. Currently, ISs are commonly recycled and used after their treatment, typically through dehydration, de-oiling, or mixing with sintering raw materials to produce sintered ore or pellet ore. Other methods for resource recycling include using these wastes to produce reduced iron powder as feedstock for powder metallurgy, or iron red pigment as a slagging agent for steelmaking [30]. However, these methods have limitations and yield low-value-added products. Additionally, the outermost layer of ISs contains Fe_2_O_3_, which is a porous material with a considerable specific surface area, large wear and impact resistance, strong adsorption capacity, and high moldability [31]. Moreover, the bandwidth of Fe_2_O_3_ is 2.2 eV [32], rendering it with photocatalytic properties. Thus, it is speculated that the preparation of photocatalytic composite materials with TiO_2_ and ISs as carriers can reduce the cost of photocatalyst composites and improve their photocatalytic activity and efficiency.

In this study, a new concept for treating liquid waste (organic pollutants) with solid waste (ISs) is proposed. The Fe_2_O_3_/TiO_2_ photocatalytic composites were synthesised using a two-step process, comprising the traditional dry process to convert ISs into highly pure Fe_2_O_3_ particles (content > 98.5 wt.%) followed by the sol-gel method. The activity of the Fe_2_O_3_/TiO_2_ photocatalytic composites in the UV degradation of a methylene blue (MB) aqueous solution (organic pollutants in simulated wastewater) was investigated, and a possible photocatalytic degradation mechanism was proposed. The Fe_2_O_3_/TiO_2_ photocatalytic composites exhibit several advantages as photocatalytic materials.

(1) Differences in research purpose and motivation: This paper focuses on the high-value utilisation of Iron Scales (ISs). According to the estimate of global crude steel production in 2022 (1.885 billion tons), iron scale production is as high as 37.7 million tons, and this study proposes to use it as a raw material to prepare Fe_2_O_3_/TiO_2_ photocatalytic composite materials and treat liquid waste (organic pollutants). This is not only an innovation in the recycling method of iron scale but also significantly improves its added value.
(2) Innovation in raw material source and preparation technology: Although the existing research mainly uses iron nitrate (Fe(NO_3_)_3_) as a precursor to prepare Fe_2_O_3_ by hydrometallurgy, this study uses a dry process to convert ISs into high-purity (greater than 98.5wt.%), porous Fe_2_O_3_ particles. This study not only realised the efficient conversion of traditional waste represented by iron scale for the first time but also provided an additional site for the adsorption of TiO_2_ nanoparticles, thereby improving the efficiency of Fe_2_O_3_ as a carrier of catalytic materials and opening up a new way of low-cost preparation.

Excellent performance of Fe_2_O_3_/TiO_2_ photocatalytic composite: Under optimised experimental conditions, the synthesised Fe_2_O_3_/TiO_2_ composite showed an efficient removal rate of 97.71% of MB organic pollutants and maintained good reuse performance after four consecutive photocatalytic cycles. This indicates that the composites synthesised in this paper have great potential as efficient photocatalysts for long-term degradation of organic pollutants.

## 2. Materials and Methods

### 2.1. Experimental Materials and Preparation Process

Tetra butyl titanate (C_16_H_36_O_4_Ti, TBT, purity = 99.0%), anhydrous ethanol (C_2_H_5_OH, purity = 99.9%), acetic acid (CH_3_COOH, purity = 99.0%), and MB (C_16_H_18_ClN_3_S, extra pure) were procured from Aladdin Biochemical Technology Co., Ltd. (Shanghai, China). Deionised water was prepared in the laboratory.

**Figure 1:**
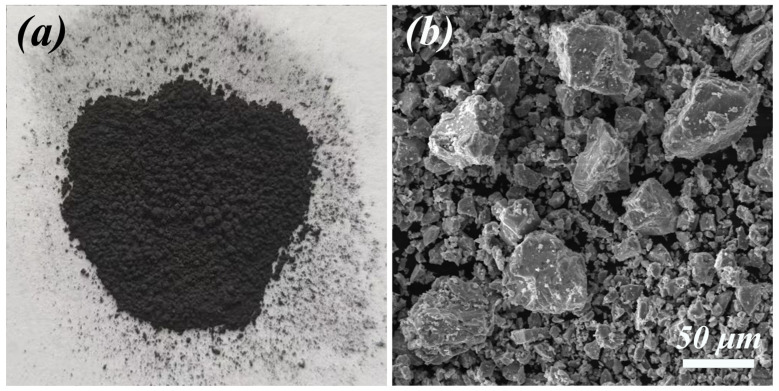
Gross morphology (**a**) and micromorphology of the iron scales (**b**).

The ISs used in this study were obtained from a cold-rolled strip production line at Angang Steel Company Limited (Anshan, China). Physical photos and scanning electron microscopy (SEM) images of the ISs are shown in Figure 1. The composition and particle size of the ISs are listed in Table 1.

Herein, Fe_2_O_3_/TiO_2_ photocatalytic composites were prepared in three stages. In the first stage, the ISs were immersed in anhydrous ethanol and ultrasonically cleaned for 30 min to eliminate surface oils. Subsequently, the as-cleaned ISs were filtered and dried in an oven at 160 °C. Subsequently, the ISs were crushed and placed in a single-temperature rotary tube furnace (RTL1200 Dia 60(OD), Nanjing Boyuntong Instrument, Nanjing, China) for oxidation and roasting. The process generated Fe_2_O_3_ particles with a purity higher than 98.5 wt.%. The oxidation of the ISs in the furnace was conducted under the following conditions: feed particle size smaller than 10 μm, roasting temperature of 800 °C, and roasting time of 4 h.

In the second stage, Fe_2_O_3_/TiO_2_ composite particles were synthesised using the sol-gel method. The beaker was filled with anhydrous ethanol (100 mL) as the solvent and acetic acid (20 mL) as the inhibitor. TBT (10 mL) was then slowly added dropwise to the solution while continuously stirring on a magnetic stirrer at a rotational speed of 500 r/min for a duration of 30 min. The as-produced Fe_2_O_3_ particles (2.5 g) were then added and stirred for 1 h until a homogenous mixture was obtained. Next, deionised water (2 mL) was slowly added using a pipette, and the solution was stirred for 2 h to form a sol. The sol was then aged for 10 h to transform it into a gel. The gel was dried in a vacuum oven at 80 °C for 24 h and then crushed using an agate mortar.

In the third stage, the resulting Fe_2_O_3_/TiO_2_ composite sample was placed in a muffle furnace and heated at a rate of 5 °C/min until the temperature reached 480 °C, after which it was calcined at a constant temperature for 2 h. Finally, the Fe_2_O_3_/TiO_2_ composite sample was cooled to 25 °C by gradually reducing the temperature of the furnace to obtain the Fe_2_O_3_/TiO_2_ photocatalytic composites.

Finally, the Fe_2_O_3_/TiO_2_ photocatalytic composite material in step 3 was added to the simulated wastewater to verify its ultraviolet degradation activity for a methylene blue (MB) aqueous solution of organic pollutants. Experimental conditions were initial MB concentration—100 mg/L, MW output power—900 W, EDLS-4, solution volume—50 mL, pH 7.

### 2.2. Performance Testing and Characterisation

The crystal structure of the produced composites was analysed using X-ray diffraction (XRD, Bruker D8 Advance, Bruker AXS, Rheinstetten, Germany) through step scanning (step = 0.02°) within a 2θ range of 10°–90°. Field emission SEM (FE-SEM, Zeiss Sigma 300, Cral Zeisis AG, Oberkochen, Germany) and energy dispersive X-ray spectroscopy (EDS, OxfordX-MAX, Cral Zissis AG, Oberkochen, Germany) were used to visualise the morphology of all the samples and to obtain their elemental composition. The elemental composition and valence states of the photocatalysts were analysed using X-ray photoelectron spectrometry (XPS, Thermo Scientific K-Alpha, Thermo Fisher Scientific, Waltham, MA, USA). Fourier transform infrared (FTIR) spectroscopy (Thermo Scientific Nicolet iS20, Thermo Fisher Scientific, Massachusetts, USA) was conducted on all samples within a wavenumber range of 4000–400 cm^−1^ over 64 scans at a resolution of 4 cm^−1^. The UV-visible (UV-vis) absorption spectra of the samples were measured using a UV-vis near-infrared spectrophotometer (Shimadzu UV-3600 Plus, Shimadzu, Kyoto, Japan). The sample was irradiated by a UV lamp (724.6 mW/cm^3^). Photoluminescence (PL) emission spectra were recorded on an Edinburg FLS1000 Photoemission Spectrometer (Edinburgh Instruments, Edinburgh, UK). The photoelectric performances of the composites were evaluated on an electrochemical workstation (CHI 760E, CH Instruments Ins, Austin, USA). Liquid chromatography-mass spectrometry (LC-MS, Shimadzu UFLC-2010 Plus, Shimadzu, Kyoto, Japan) was used to detect the fragments (intermediates) of the MB degradation. Specifically, in this study, the main wavelength range of the xenon lamp used is concentrated in the region of about 510 nm; At the same time, in order to effectively exclude the influence of ultraviolet light, a filter is set at 400 nm.

Herein, MB solutions (40 mL) with mass concentrations of 10, 20, 30, and 40 mg/L were prepared as simulated contaminant solutions. Subsequently, the as-synthesised Fe_2_O_3_/TiO_2_ photocatalytic composites (100 mg) were added to each solution. The visible-light catalytic performance of the photocatalysts was analysed using a xenon light source (PLS-SXE300/300UV, PerfectLight, Beijing, China) to simulate visible light. To evaluate the photodegradation process, four experiments with different times (30, 60, 90, and 120 min) were conducted. Before each test, the mixtures were ultrasonically shaken and mixed under dark conditions for 15 min. After the photodegradation process for each experimental group, the mixture was centrifuged and filtered to collect the supernatant for analysis. This liquid sample was then analysed for absorbance using a UV-visible spectrophotometer (LH-3BA, Lianhuakeji, Beijing, China) at a detection wavelength of 664 nm. Owing to the linear relationship between the concentration of the MB solution and absorbance, the decolourisation rate is equivalent to the photodegradation rate. The photodegradation rate (D) was calculated using Equations (1) and (2) [33], as follows:

D = (A_0_ − A)/A_0_ × 100%, (1)

ln(A_0_/A) = kt, (2)

Here, A_0_ (mg/L) and A (mg/L) are the absorbance values of the undegraded and degraded MB solutions, respectively. For comparison, photodegradation experiments were conducted using pure anatase TiO_2_ materials and the Fe_2_O_3_ produced in the initial step under the same conditions.

Four cycles of photocatalysis experiments were performed under optimal conditions to examine the reusability of the synthesised Fe_2_O_3_/TiO_2_ photocatalytic composites. The regeneration of the pregnant Fe_2_O_3_/TiO_2_ was conducted by submerging the composite into a NaOH solution (0.1 mol/L) and stirring for 1 h. It was then repeatedly washed using deionised water and ethanol. The regenerated composite was subsequently dried in an oven at 40 °C until constant weight.

## 3. Results

### 3.1. Characterization of Fe_2_O_3_/TiO_2_ Photocatalytic Composites

#### 3.1.1. XRD Patterns

**Figure 2:**
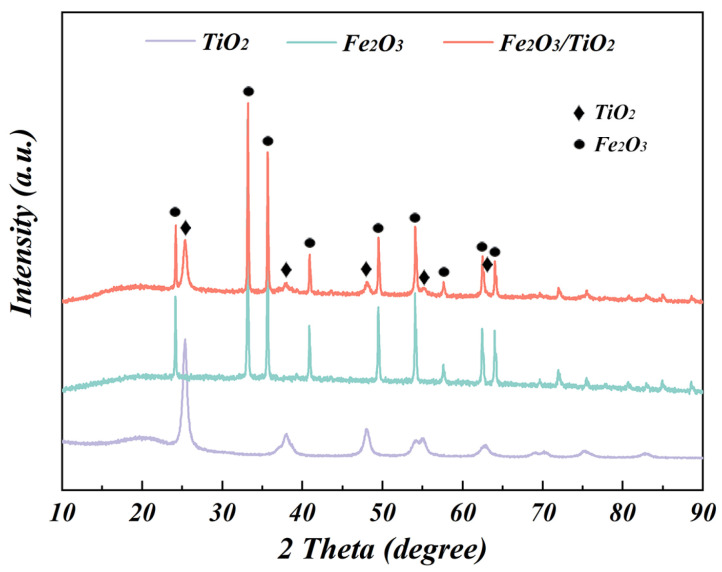
X-ray diffraction patterns of the unloaded TiO_2_ particles, Fe_2_O_3_ particles and Fe_2_O_3_/TiO_2_ photocatalytic composites.

Figure 2 depicts the XRD patterns of three samples: the unloaded TiO_2_ particles, Fe_2_O_3_ particles obtained through the oxidation roasting of ISs, and as-synthesised Fe_2_O_3_/TiO_2_ photocatalytic composites. The diffraction peaks of the unloaded TiO_2_ particles are observed at 25.4°, 37.9°, 48.0°, 54.0°, and 62.8°, which respectively correspond to the (101), (004), (200), (105) and (204) crystal planes of the anatase phase (ICOD card No. 01-073-1764). The Fe_2_O_3_ particles exhibit distinct diffraction peaks at 2θ values of 24.2°, 33.2°, 35.7°, 40.9°, 49.5°, 54.1°, 57.6°, 62.5°, and 64.0°, which are, respectively, associated with the (012), (104), (110), (113), (024), (116), (018), (214) and (300) crystal planes of the hematite-type Fe_2_O_3_ (ICOD card No. 01-073-2234). Moreover, the Fe_2_O_3_/TiO_2_ photocatalytic composites maintain the diffraction peaks of hematite Fe_2_O_3_ and the characteristic peaks of anatase TiO_2_. Although the intensity of the TiO_2_ characteristic peaks in the Fe_2_O_3_/TiO_2_ photocatalytic composites is slightly weaker than that of the peaks of the unloaded TiO_2_ particles, the overall peak shape of TiO_2_ remains unchanged, suggesting that the presence of Fe_2_O_3_ weakens the lattice signal of TiO_2_. However, the crystallinity and structural integrity of the as-synthesised Fe_2_O_3_/TiO_2_ photocatalytic composites are enhanced. To examine the effect of the Fe_2_O_3_ loading on the crystal structure and grain size of the TiO_2_ particles in the Fe_2_O_3_/TiO_2_ photocatalytic composites, the XRD results of the unloaded TiO_2_ particles were compared to those of the as-synthesised Fe_2_O_3_/TiO_2_ photocatalytic composites (Supplementary Figure S1). Supplementary Table S1 provides detailed information on the crystal lattice constants. The low values of the weighted peak residual variance factor (Rwp) indicate excellent agreement between the calculated and experimental results. The refinement of the XRD results indicates that the lattice parameters of the anatase TiO_2_ in the Fe_2_O_3_/TiO_2_ photocatalytic composites are a = 3.788 Å, c = 9.500 Å, and α = β = γ = 90°, which closely match the values of the unloaded anatase TiO_2_ particles (a = 3.790 Å and c = 9.491 Å). This slight variation confirms that the crystal structure of anatase TiO_2_ is maintained. Furthermore, the lattice parameters of the Fe_2_O_3_ carrier were also determined to be a = 5.034 Å, c = 13.747 Å, α = β = 90°, and γ = 120°. The grain size could be calculated using Scherrer’s equation based on the determined lattice parameters as follows (Equation (3)) [34]:

D = kλ/βcosθ,(3)

here, D is the grain size, k is Scherrer’s constant, λ is the X-ray wavelength, β is the full-width half maximum (FWHM) of the XRD peaks, and θ is the peak angle. The calculations show that the grain size of the pure unloaded anatase TiO_2_ is 31.81 nm, whereas the average grain size of TiO_2_ in the Fe_2_O_3_/TiO_2_ photocatalytic composites is 31.74 nm. The above XRD results demonstrate that the Fe_2_O_3_ loading does not considerably affect the crystal structure or grain size of anatase TiO_2_.

#### 3.1.2. SEM-EDS Analysis

**Figure 3:**
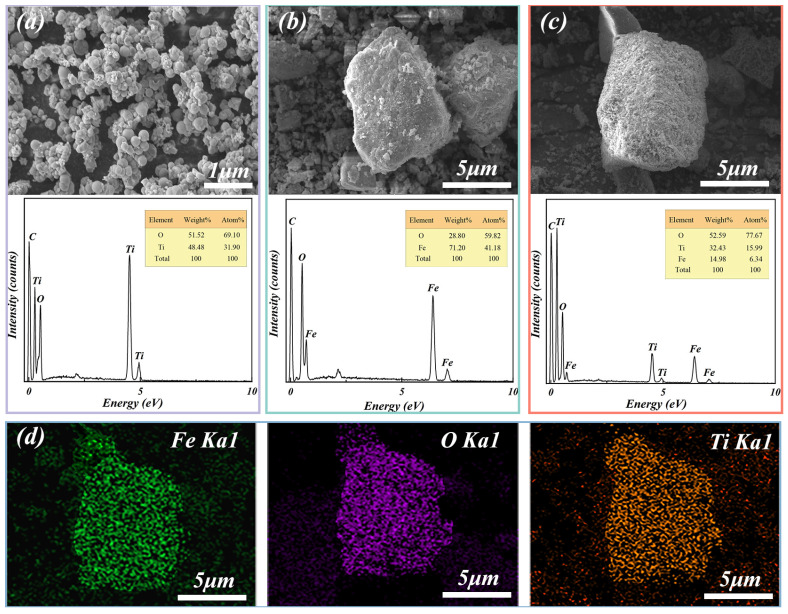
Scanning electron microscopy images and energy dispersive X-ray spectroscopy results of the (**a**) unloaded TiO_2_ particles, (**b**) Fe_2_O_3_ particles and (**c**) Fe_2_O_3_/TiO_2_ photocatalytic composites. (**d**) Elemental mapping distribution of Fe, O and Ti on the surface of the Fe_2_O_3_/TiO_2_ photocatalytic composites.

Figure 3 illustrates the SEM images and EDS results of the unloaded TiO_2_ particles, Fe_2_O_3_ particles obtained through oxidation roasting of ISs, and as-synthesised Fe_2_O_3_/TiO_2_ photocatalytic composites. As depicted in Figure 3a, the unloaded TiO_2_ particles prepared using the combined sol-gel/calcination method exhibit agglomeration with an average particle size of approximately 200 nm. The EDS analysis confirms the presence of Ti and O elements only. As shown in Figure 3b, the Fe_2_O_3_ particles indicate an irregular shape and a rough surface with a diameter of less than 10 μm. The EDS measurements indicate that the Fe and O contents are 71.20 wt.% and 28.80 wt.%, respectively, which is in good agreement with the theoretical values of the elements in Fe_2_O_3_. Figure 3c illustrates the even loading of the TiO_2_ nanoparticles on the surface of the Fe_2_O_3_ particles, resulting in a decrease in TiO_2_ aggregation. This phenomenon follows the Ostwald ripening mechanism [35], where the smaller TiO_2_ crystal nuclei converge onto the larger Fe_2_O_3_ particles, gradually forming TiO_2_ crystal cells until a complete and uniform coating of Fe_2_O_3_ particles occurs, indicating the formation of Fe_2_O_3_/TiO_2_ photocatalytic composites with a core-shell structure. Additionally, the EDS analysis of the Fe_2_O_3_/TiO_2_ photocatalytic composites reveals strong signals of Ti, O and Fe elements. Compared to the Fe_2_O_3_ particles, the elemental Ti and O contents in the composites are considerably higher (32.43 wt.% and 52.59 wt.%, respectively), whereas their Fe content is lower (14.98 wt.%). Furthermore, EDS element mapping (Figure 3d) further confirms the uniform distribution of Fe, O, and Ti on the surface of the Fe_2_O_3_/TiO_2_ photocatalytic composites. In conclusion, the XRD, SEM, and EDS results demonstrate that ISs can be transformed into highly pure and porous Fe_2_O_3_ particles through oxidation roasting. This process provides ISs with additional sites for the adsorption and attachment of nanoparticles, transforming them into effective carriers for TiO_2_-based catalysts. In the sol-gel process, TiO_2_ is loaded onto the Fe_2_O_3_ particles, resulting in the formation of Fe_2_O_3_/TiO_2_ photocatalytic composites, in which the Fe_2_O_3_ particles and anatase TiO_2_ nanoparticles act as the core and shell, respectively.

#### 3.1.3. FTIR Analysis

**Figure 4:**
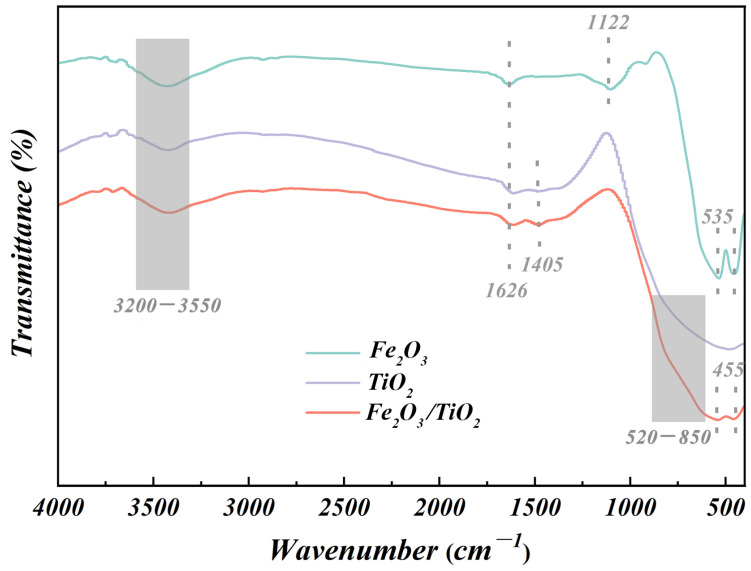
Fourier transforms infrared spectra of the Fe_2_O_3_ particles, unloaded TiO_2_ particles and Fe_2_O_3_/TiO_2_ photocatalytic composites.

The chemical structure and functional groups of the synthesised photocatalytic composites were analysed using FTIR. Figure 4 depicts the FTIR spectra of the Fe_2_O_3_ particles, unloaded TiO_2_ particles, and as-synthesised Fe_2_O_3_/TiO_2_ photocatalytic composites. In these three cases, the broad region within 3200–3550 cm^−1^ corresponds to the stretching vibrations of the hydroxyl groups. Moreover, a characteristic peak is observed near 1626 cm^−1^, which corresponds to the bending vibration of the active O-H bond, indicating the presence of adsorbed free water molecules in the structures of the three species [36]. These hydroxyl groups and the adsorbed water actively participate in the photocatalytic reaction, reacting with the excited holes on the catalyst surface to generate •OH, which effectively oxidises and degrades organic pollutants. The FTIR spectra of the Fe_2_O_3_ particles exhibit distinct peaks at 455, 535, and 1122 cm^−1^, corresponding to the Fe-O bond vibration (metal-oxygen bond), bending vibration, and tensile vibration of the α-Fe_2_O_3_ structure, respectively [37]. In the FTIR spectrum of the unloaded TiO_2_ particles, the peak observed at 1405 cm^−1^ corresponds to the stretching vibration of the Ti-O-Ti bond, and the stretching vibrations of Ti-O-Ti and O-Ti-O cause a broad peak at 520–850 cm^−1^ [38]. The characteristic peaks at 455, 535, and 1405 cm^−1^ in the FTIR spectrum of the Fe_2_O_3_/TiO_2_ photocatalytic composites confirm the presence of iron oxides and the successful deposition of anatase TiO_2_ crystals on Fe_2_O_3_. However, a comparison between the Fe-O vibration peaks in the curves of the Fe_2_O_3_ particles and Fe_2_O_3_/TiO_2_ photocatalytic composites indicates that the positions of these peaks remain unchanged. The experimental results indicate the absence of a chemical bond between the TiO_2_ components and Fe_2_O_3_ carriers in the Fe_2_O_3_/TiO_2_ photocatalytic composites, which is in good agreement with the findings obtained from the XRD analysis.

#### 3.1.4. XPS Analysis

**Figure 5:**
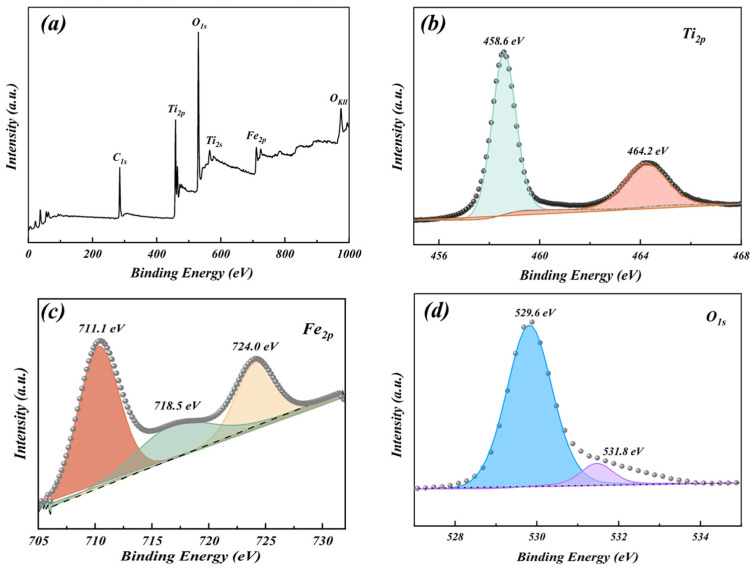
X-ray photoelectron spectra of the Fe_2_O_3_/TiO_2_ photocatalytic composites: (**a**) full scan survey of all elements and high-resolution spectra of (**b**) Ti_2p_, (**c**) Fe_2p_ and (**d**) O_1s_.

The elemental composition and chemical bonding of the Fe_2_O_3_/TiO_2_ photocatalytic composites were analysed using XPS. Figure 5a depicts the XPS full spectrum of the Fe_2_O_3_/TiO_2_ photocatalytic composites, and Figure 5b–d shows the high-resolution XPS diagrams of the Ti, Fe, and O elements, respectively, in the composites. As depicted in Figure 5a, the Fe_2_O_3_/TiO_2_ photocatalytic composites also contain a small amount of C in addition to Fe, Ti, and O. However, the photocatalytic composites do not naturally contain C, and the C_1s_ peak observed at 284.8 eV can be attributed to the introduction of C during XPS testing. The peaks observed at 458.6, and 464.2 eV (Figure 5b) correspond to the split electrons in the Ti_2p3/2_ [39] and Ti_2p1/2_ [40] spin orbitals, respectively, thus confirming the presence of Ti^4+^ in the Fe_2_O_3_/TiO_2_ photocatalytic composites. Similarly, the peak observed at 711.1 eV, and the satellite peak at 723.8 eV [41] (Figure 5c) correspond to the Fe_2p3/2_ orbital, whereas the peak observed at 724.0 eV corresponds to the Fe_2p1/2_ orbital [42], confirming the presence of Fe^3+^ in the Fe_2_O_3_/TiO_2_ photocatalytic composites. Furthermore, the binding energy peak observed at 529.6 eV (Figure 5d) represents a characteristic absorption peak of the lattice oxygen in metal oxides [43], indicating the presence of O in the form of O^2−^. The peak observed at 531.8 eV may correspond to chemisorbed oxygen, possibly resulting from the presence of adsorbed water molecules in the form of hydroxyl (−OH) on the surface of the composites [44]. These findings are in good agreement with the results obtained from EDS and XRD analyses, confirming that the composite catalyst primarily consists of Fe_2_O_3_ and TiO_2_ without any additional impurities.

#### 3.1.5. UV-Vis Diffuse Reflection Spectroscopy (DRS) Analysis

**Figure 6:**
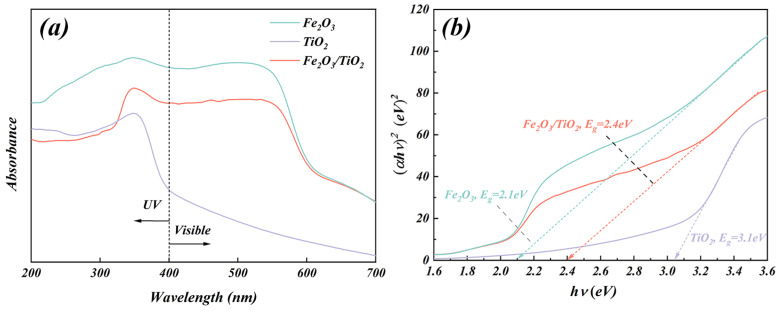
(**a**) Ultraviolet-visible diffuse reflection spectroscopy of the Fe_2_O_3_ particles, unloaded TiO_2_ particles and Fe_2_O_3_/TiO_2_ photocatalytic composites as well as (**b**) their corresponding (αhν)^2^ vs. hν curves.

The UV-vis DRS absorption spectra of the Fe_2_O_3_ particles, unloaded TiO_2_ particles, and Fe_2_O_3_/TiO_2_ photocatalytic composites are shown in Figure 6a. The absorption wavelength of the unloaded anatase TiO_2_ particles is observed in the UV region (wavelength < 400 nm), and almost no absorption peak is observed in the non-UV region (wavelength > 400 nm), which confirms the low visible-light utilisation limitation of single anatase TiO_2_. Conversely, the absorption band edges of the Fe_2_O_3_ particles and Fe_2_O_3_/TiO_2_ photocatalytic composites exceed 400 nm, and they exhibit clear absorption peaks in both the UV and visible regions, indicating that they can absorb photons in the visible region. The photocatalyst exhibits a significant redshift due to the release of oxygen in its structure [45]. Furthermore, the bandgap energy can be obtained from the UV-vis DRS spectra, whereas the optical bandgap values (E_g_) of the Fe_2_O_3_ particles, unloaded TiO_2_ particles, and Fe_2_O_3_/TiO_2_ photocatalytic composites are estimated using Equation (4) as follows [46]:

αhν = A(hν − E_g_)^n/2^, (4)

Here, α, h, ν, E_g,_ and A are the absorption coefficient, Planck’s constant, optical frequency, optical bandgap energy, and a constant, respectively. The n index depends on the electronic transition of the semiconductor; for direct- and indirect-bandgap semiconductors, n = 1 and n = 4, respectively [47]. Rutile has both direct and indirect bandgaps, whereas anatase only has an indirect bandgap [48]. Assuming that Fe_2_O_3_ and Fe_2_O_3_/TiO_2_ are indirect semiconductors, their bandgap energy can be calculated from the curve representing the relationship between (αhν)^1/2^ and photon energy (hν) (Figure 6b). The E_g_ of the Fe_2_O_3_ particles unloaded TiO_2_ particles, and Fe_2_O_3_/TiO_2_ photocatalytic composites are 2.1, 3.1 and 2.4 eV, respectively. The shifts in the bandgap energy of the Fe_2_O_3_/TiO_2_ photocatalytic composites also prove that the photoelectron-hole pair can be generated more easily after the Fe_2_O_3_ loading with shorter light absorption and electron transport paths, increasing the catalytic reaction rate and photocatalytic activity. Several previous studies [49,50,51] have reported that the valence band (VB) and conduction band (CB) positions in photocatalysts can be calculated based on E_g_ according to the following empirical equations (Equations (5) and (6)) [51]:

E_CB_ = X − E^e^ − 0.5E_g_ (5)

E_VB_ = E_CB_ + E_g_ (6)

Here, E^e^ is the energy of free electrons on the hydrogen scale (4.5 eV), X is the geometric mean of the electronegativity of the constituent atoms (the X values of Fe_2_O_3_ and TiO_2_ are 5.88 and 2.33 eV, respectively). E_CB_ and E_VB_ are calculated based on the UV-vis diffuse reflectance absorption spectra, and the results are presented in Table 2.

#### 3.1.6. PL Analysis

**Figure 7:**
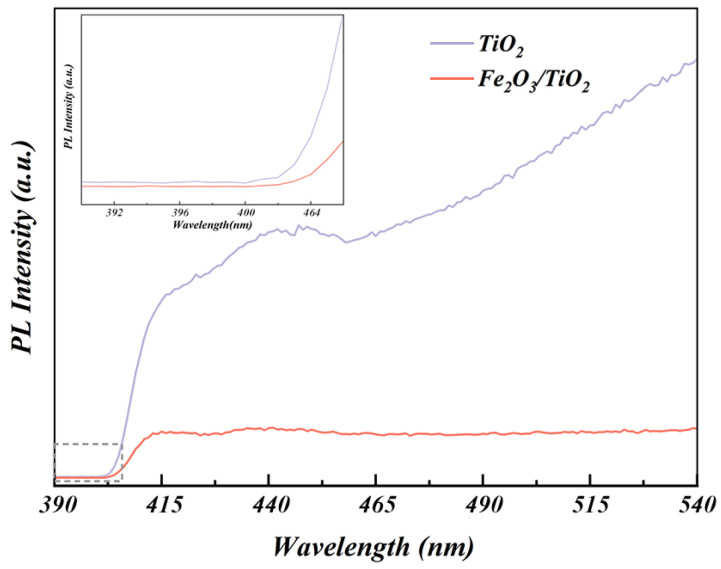
Photoluminescence spectroscopy of the unloaded TiO_2_ particles and Fe_2_O_3_/TiO_2_ photocatalytic composites.

The electron-hole recombination rates in the unloaded TiO_2_ particles and Fe_2_O_3_/TiO_2_ photocatalytic composites were evaluated using PL spectroscopy (Figure 7). The excitation source is a 405 nm laser system. A reduction in the PL peak intensity reflects a decrease in the recombination rate between the photogenerated electrons and holes. Thus, the low peak intensity in the PL spectrum indicates a high rate of electron-hole separation, indicating high photocatalytic activity. Figure 7 clearly demonstrates that the Fe_2_O_3_/TiO_2_ photocatalytic composites exhibit a significant decrease in the PL peak intensity compared with the unloaded TiO_2_ particles, signifying a substantial improvement in the separation efficiency of the electron-hole pairs. This favourable outcome can be attributed to the presence of Fe_2_O_3_, which induces rapid charge separation and transfer while effectively inhibiting the electron-hole recombination [52,53].

#### 3.1.7. Photocurrent Density Analysis

**Figure 8:**
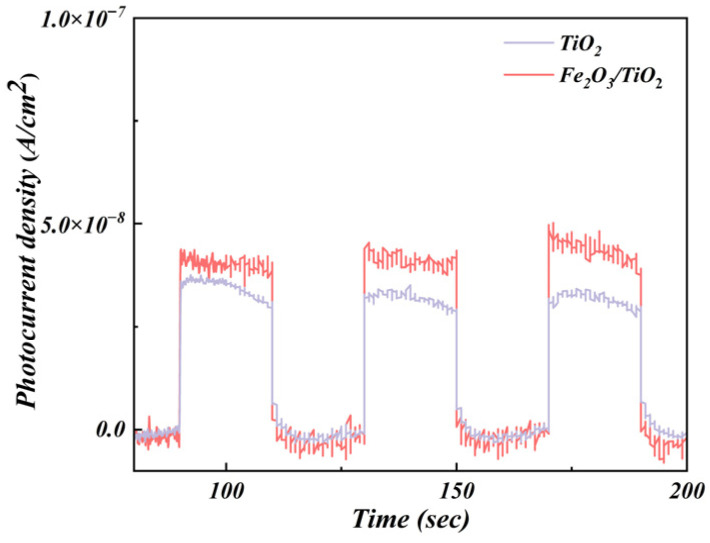
Photocurrent density of the unloaded TiO_2_ particles and Fe_2_O_3_/TiO_2_ photocatalytic composites.

The effective transfer of charges and separation of the electron-hole pairs directly affect the catalytic activity of photocatalysts. Photocurrent measurements were conducted to evaluate the capability of the synthesised materials to separate the photoexcited electron-hole pairs. Figure 8 depicts the photocurrent density of the unloaded TiO_2_ particles and Fe_2_O_3_/TiO_2_ photocatalytic composites over three switching cycles under simulated sunlight. After light irradiation, the photocurrent rapidly increases and reaches a steady state. When the light is switched off, the photocurrent rapidly returns to its dark-current state. As shown in Figure 8, the current density increases upon illumination and promptly decreases when the illumination ceases, eventually stabilising at around zero. The Fe_2_O_3_/TiO_2_ photocatalytic composites show favourable cyclic performance over numerous cycles, confirming the relative stability of their photocurrent properties. Moreover, this phenomenon suggests that photocurrent generation originates from the intrinsic behaviour of the photocurrent itself, enhancing the transport of the carriers. Compared to the unloaded TiO_2_ particles, the Fe_2_O_3_/TiO_2_ photocatalytic composites exhibit a higher photocurrent response (an increase higher than 15.6%). This indicates that the presence of Fe_2_O_3_ promotes the separation of the electron-hole pairs in TiO_2_, enhances the transfer rate of the photogenerated carriers, prolongs the lifetime of the photogenerated electrons and holes, and improves the photoresponse performance of TiO_2_ [54]. Consequently, this enhancement is anticipated to improve the photodegradation reaction of organic pollutants.

### 3.2. Analysis of the Photodegradation of MB

#### 3.2.1. Effect of Reaction Time

**Figure 9:**
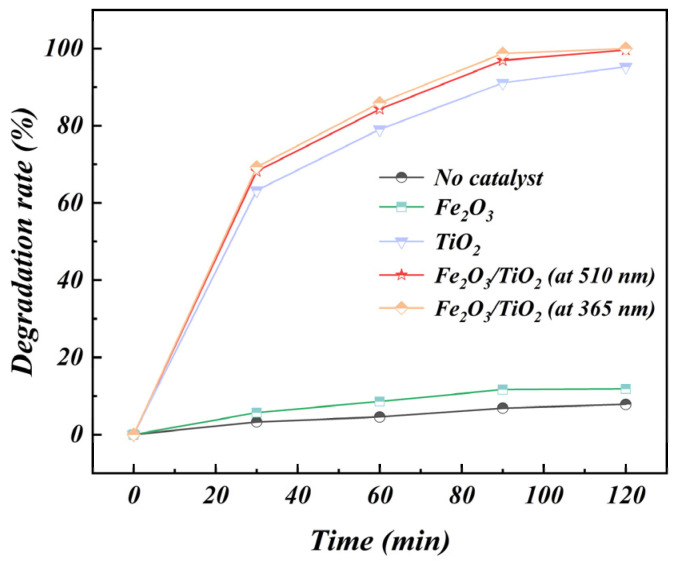
Photocatalytic activity of the Fe_2_O_3_ particles unloaded TiO_2_ particles and Fe_2_O_3_/TiO_2_ photocatalytic composites under simulated visible-light irradiation and the photocatalytic activity of Fe_2_O_3_/TiO_2_ photocatalytic composites under ultraviolet irradiation (Experimental conditions: composite mass = 100 mg, solution volume = 40 mL, initial concentration = 30 mg/L, ultraviolet light irradiation power = 400 W, pH of methylene blue = 7 and time = 60 min).

The photocatalytic activity of the Fe_2_O_3_ particles unloaded TiO_2_ particles and Fe_2_O_3_/TiO_2_ photocatalytic composites was investigated at an MB dye concentration of 30 mg/L. The MB solution was exposed to visible light, simulated using a xenon lamp, for 120 min. To ensure the suitability of the developed photocatalyst for future practical applications, its performance was evaluated under neutral pH conditions, and the results are illustrated in Figure 9. The figure shows that the MB dye exhibits a low self-degradation rate and good chemical stability. When no catalytic material is added, i.e., with only Fe_2_O_3_ particles present, the MB concentration barely changes. However, the samples of the unloaded TiO_2_ particles and Fe_2_O_3_/TiO_2_ photocatalytic composites exhibit faster removal rates over the first 90 min, effectively decomposing MB dye molecules into CO_2_ and H_2_O with faster removal rates (91.06% and 96.91%, respectively). In the first 15 min, the composite catalyst has a high photodegradation rate. This is due to the high initial concentration of MB. The strong driving force caused by the high concentration difference promotes the adsorption of MB on the material, thereby accelerating the subsequent photodegradation reaction rate. With the extension of time, the concentration of MB adsorbed on the Fe_2_O_3_/TiO_2_ composite catalyst gradually decreases, and the adsorption-desorption on the Fe_2_O_3_/TiO_2_ composite catalyst gradually reaches equilibrium. This phenomenon can be attributed to the presence of holes and electrons at the adsorption site on the catalyst surface, which generate superoxide anions (O^2−^) and hydroxyl radicals (OH) upon UV irradiation. Consequently, there is an accelerated initial photodegradation of the dye contaminants. Nevertheless, over time, the dye molecules occupy the active sites, and the driving force for adsorption decreases with the decrease in the concentration of the residual dye, ultimately impeding the interaction between the oxidising species and free dye molecules and leading to a decrease in the degradation rate.

#### 3.2.2. Effect of Initial Concentration

**Figure 10:**
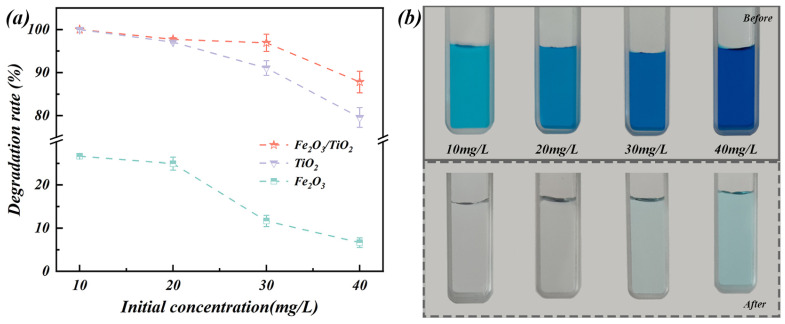
(**a**) Effect of the initial concentrations on the photocatalytic removal of MB by the Fe_2_O_3_ particles, unloaded TiO_2_ particles and Fe_2_O_3_/TiO_2_ photocatalytic composites. (**b**) Colour difference in each of four MB solutions with different concentrations before and after the degradation reaction by the Fe_2_O_3_/TiO_2_ photocatalytic composites for 2 h.

The initial concentration of the pollutant plays a crucial role in determining the adsorption driving force, which subsequently affects the molecules attached to the surface of the material. The degradation of MB by the Fe_2_O_3_ particles unloaded TiO_2_ particles, and Fe_2_O_3_/TiO_2_ photocatalytic composites was studied at an MB initial concentration of 10–40 mg/L (Figure 10a). At low initial concentrations (10 and 20 mg/L), the degradation efficiency of MB by the Fe_2_O_3_/TiO_2_ photocatalytic composites is comparable to that of the unloaded TiO_2_ particles. However, as the initial concentration of the MB solution increases to 30 mg/L, a noticeable difference is observed in the degradation rates of the Fe_2_O_3_/TiO_2_ photocatalytic composites and unloaded TiO_2_ particles. The photodegradation of the Fe_2_O_3_/TiO_2_ photocatalytic composites is considerably superior to that of the unloaded TiO_2_ particles. With an increase in the MB initial concentration from 10–40 mg/L, the removal rate induced by the Fe_2_O_3_/TiO_2_ photocatalytic composites decreases from 100% to 87.81%. This behaviour is characteristic of the photocatalytic process because the active sites occupied by pollutants obstruct the contact between the free dye molecules and the Fe_2_O_3_/TiO_2_ photocatalytic composites, resulting in a low dye-removal rate [55]. In addition, the high concentration of the MB dye increases the colour of the solution, which hinders the penetration of the UV light from reaching the surface of the material, limiting the generation of the photo-induced free radicals required for the degradation process [56]. Moreover, the colour difference in each of the four MB solutions with different concentrations was compared before and after the degradation reaction by the Fe_2_O_3_/TiO_2_ photocatalytic composites for 2 h (Figure 10b).

#### 3.2.3. Reaction Kinetics Study

**Figure 11:**
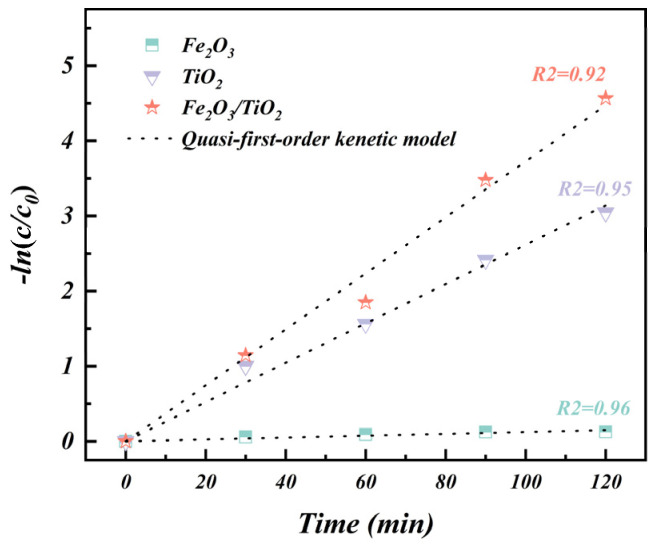
Quasi-first-order kinetic fitting curves of the MB photodegradation reaction.

The reaction rate is a crucial parameter for evaluating the removal rate and reaction mechanism of MB degradation. In this study, the kinetics of the MB photodegradation reaction was investigated using quasi-first-order kinetic models [57,58,59]. The calculated values of k for the different catalysts are presented in Figure 11. The MB degradation rate constant of the Fe_2_O_3_/TiO_2_ photocatalytic composites (k = 0.038 min^−1^) is 1.5 times higher than that of the unloaded TiO_2_ particles (k = 0.026 min^−1^) and 38 times greater than that of the Fe_2_O_3_ particles (k = 0.001 min^−1^). Thus, from the perspective of the photodegradation kinetics, the Fe_2_O_3_/TiO_2_ photocatalytic composites exhibit the highest catalytic effect for MB degradation.

#### 3.2.4. Material Reusability

**Figure 12:**
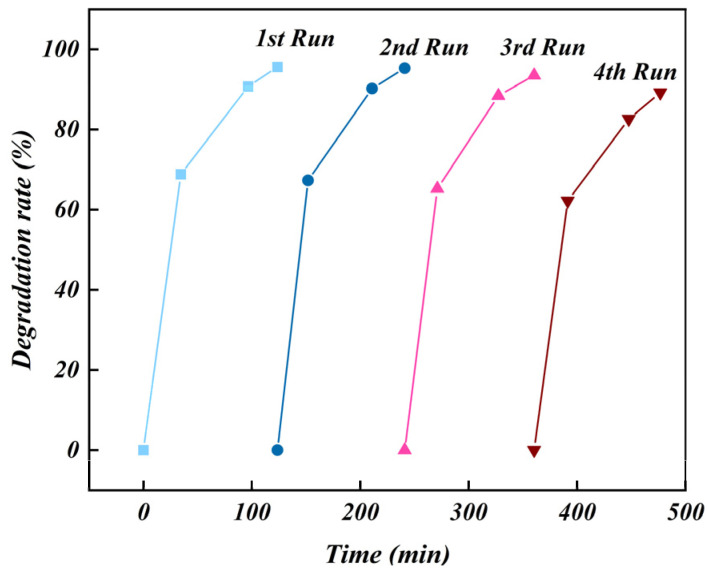
Cyclic tests of the Fe_2_O_3_/TiO_2_ photocatalytic composites used for treating methylene blue dye pollutants. (Experimental conditions: composite mass = 100 mg, solution volume = 40 mL, initial concentration of methylene blue = 20 mg/L, ultraviolet light irradiation power = 400 W, pH of methylene blue solution = 7 and time = 90 min).

To investigate the stability and recyclability of the Fe_2_O_3_/TiO_2_ photocatalytic composites, they were regenerated under optimal conditions for photodegradation, and cyclic tests were then conducted. The experimental findings are presented in Figure 12. During the cyclic test, the photocatalytic activity of the Fe_2_O_3_/TiO_2_ photocatalytic composites remained consistently high, with only a slight decrease, indicating its remarkable stability. The reduction in its degradation efficiency can be attributed to the presence of adsorbed intermediates and residual organic matter that remain incompletely degraded during the reaction, occupying active sites on the composite material and impeding its interaction with free dye molecules in the solution [60]. These results reveal that the synthesised Fe_2_O_3_/TiO_2_ photocatalytic composites have excellent stability and recyclability, confirming its viability as a reusable photocatalyst for long-term use in the degradation of organic pollutants.

There are four main reasons for the excellent catalytic performance of the prepared Fe_2_O_3_/TiO_2_ photocatalytic composites. First, the narrow bandgap of Fe_2_O_3_ promotes the utilisation of visible light. Second, the good adsorption behaviour of organic substances on porous Fe_2_O_3_ helps bring the reactants close to the oxidation free radicals generated by light, improving the reaction kinetics. Third, the Fe_2_O_3_ loading facilitates the generation of photoelectron-hole pairs and reduces the light absorption and electron transport path, increasing the rate of the catalytic reaction and photocatalytic activity. Finally, Fe_2_O_3_ is an excellent electron acceptor, which enhances electron transfer through local electrostatic fields. Moreover, the presence of Fe_2_O_3_ promotes the separation of electron-hole pairs within TiO_2_. In addition, Ti^4+^ provides a sub-bandgap in the composite material, facilitating the electron transition from the VB to the CB, whereas the oxygen vacancy acts as a hole trap, effectively inhibiting electron-hole recombination. These effects increase the lifetime of photogenerated electrons and holes [13,20,61]. Thus, the synergistic effects of each component of the Fe_2_O_3_/TiO_2_ photocatalytic composites enhance their adsorption, light utilisation and charge separation performance.

### 3.3. Degradation Pathways of MB Dye

**Figure 13:**
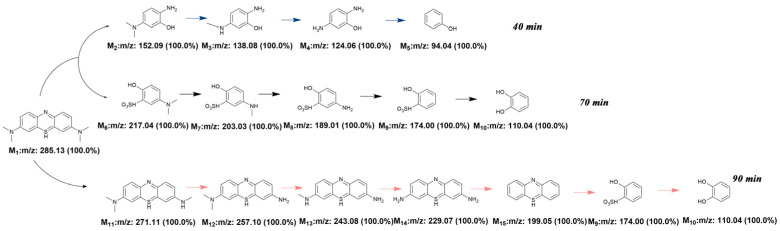
Proposed degradation pathway of methylene blue under ultraviolet light irradiation.

The intermediates generated during the degradation process of pollutants were analysed using LC-MS, and the potential pathway of the mineralisation system for the MB dye is illustrated in Figure 13. During the degradation of MB, the S-Cl bond is initially cleaved, resulting in the formation of chloride ions (Cl^−^). The remaining portion then decomposes through two distinct mechanisms. First, under the effect of the active substances, the N-CH_3_ bond is broken, leading to considerable decolourisation through demethylation. Next, the C-S bond is oxidised, forming a new chemical bond known as sulfonyl (S = O), which then detaches from the benzene ring and further oxidises to yield sulfate ions (SO_4_^2−^). Simultaneously, the -NH_2_ chemical bond is also broken, increasing the formation of ammonium ions (NH^4+^). These ammonium ions are further oxidised to generate nitrite ions (NO^2−^) and nitrate ions (NO^3−^), which eventually undergo complete mineralisation to CO_2_ and H_2_O [62].

## 4. Discussion

**Figure 14:**
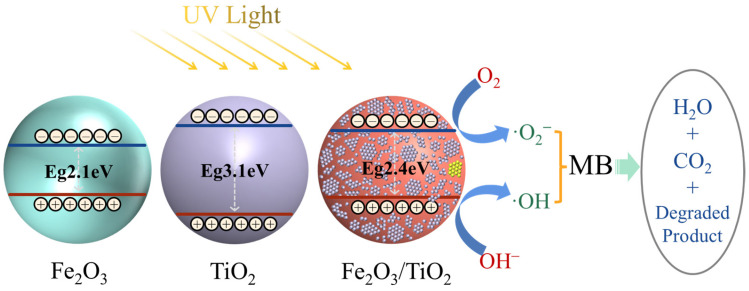
Degradation mechanism of the methylene blue solution under ultraviolet light.

Figure 14 illustrates the band structure of the Fe_2_O_3_ particles, unloaded TiO_2_ particles, and Fe_2_O_3_/TiO_2_ photocatalytic composites, as well as the degradation mechanism of the MB solution under UV light. Consider the Fe_2_O_3_/TiO_2_ photocatalytic composites as an example. When photons carrying sufficient energy equal to or greater than the bandgap width of the material are absorbed by the surface of the material under UV irradiation, photogenerated electrons (e_cb_^−^) and photogenerated holes (h_vb_^+^) are generated on the catalyst surface. Subsequently, e_cb_^−^ undergoes an in situ transition from the VB to the CB of the Fe_2_O_3_/TiO_2_ photocatalytic composites, whereas h_vb_^+^ remains in the VB. The negatively charged e_cb_^−^ is trapped by O_2_, forming a superoxide anion (•O_2_^−^), whereas the positively charged h_vb_^+^ reacts with the OH^−^ group on the catalyst surface, leading to the formation of a highly reactive hydroxyl radical (•OH). This reaction also helps prevent the recombination of h_vb_^+^ and e_cb_^−^. The resulting active species, including •O_2_^−^ and •OH, show a strong redox capacity, enabling them to effectively degrade dyes, such as MB, into environmentally benign products, such as H_2_O and CO_2_. Moreover, h_vb_^+^ can directly react with MB to produce H_2_O and CO_2_ [63,64]. According to research reports, TiO_2_ (titanium oxide type): VB max: (+ 3.0 eV), CB min: (−0.5 eV), band gap is about 3.1 eV [65]; Fe_2_O_3_ (Hematite type): VB max: (+ 2.2 eV), CB min: (−0.5 eV), bandgap about 2.1 eV [66]. The following are the primary reactions involved in the photodegradation process [67]:

Fe_2_O_3_/TiO_2_ + hv(E_g_ ≥ 2.4 eV) → Fe_2_O_3_/TiO_2_(h_vb_^+^ + e_cb_^−^) (7)

e_cb_^−^ + O_2_ → •O_2_^−^ (8)

h_vb_^+^ + OH^−^ → •OH + H^+^ (9)

•OH + •O_2_^−^ + MB → Degradation products + H_2_O + CO_2_ (10)

h_vb_^+^ + MB → Degradation products + H_2_O + CO_2_ (11)

## 5. Conclusions

This study proposed the use of IS solid waste as a raw material for preparing photocatalytic composites to treat liquid waste (organic pollutants). The novel Fe_2_O_3_/TiO_2_ photocatalytic composites were synthesised by traditional oxidation roasting combined with the sol-gel method. The Fe_2_O_3_/TiO_2_ photocatalytic composites were employed to effectively remove MB under UV light. The structural and reaction characteristics of the composites were thoroughly assessed using various techniques. The main findings of this study are as follows:

(1) The oxidation roasting process successfully transforms ISs into highly pure, porous Fe_2_O_3_ particles, providing additional sites for the adsorption of the TiO_2_ nanoparticles on the composites. This enables the Fe_2_O_3_ particles to act as effective carriers for the catalytic materials. In the sol-gel process, a photocatalytic composite comprising Fe_2_O_3_ particles and anatase TiO_2_ nanoparticles as the core and shell, respectively, is formed. The loading of Fe_2_O_3_ barely affects the crystal structure and grain size of anatase TiO_2_.
(2) The Fe_2_O_3_/TiO_2_ photocatalytic composites exhibit outstanding removal performance of MB organic pollutants. Under optimised experimental conditions, the MB removal rate reaches an impressive 97.71% with good reusability after four photocatalytic cycles. Additionally, during dye degradation, the reaction process follows a quasi-first-order kinetic model with a rate constant of 0.038 min^−1^, which is 1.5 times higher than that of the unloaded TiO_2_ particles (k = 0.026 min^−1^) and 38 times higher than that of the Fe_2_O_3_ particles (k = 0.001 min^−1^).
(3) The incorporation of Fe_2_O_3_ as a support enhances the separation of the electron-hole pairs in TiO_2_ and improves the transfer rate of the photogenerated carriers. Furthermore, it extends the lifetime of the photogenerated electrons and holes. The presence of Fe_2_O_3_ further reduces the bandgap (2.4 eV) of the Fe_2_O_3_/TiO_2_ photocatalytic composites, considerably contributing to its high photocatalytic activity.

In summary, the Fe_2_O_3_/TiO_2_ photocatalytic composites are an efficient green photocatalyst that uses high-value-added metallurgical solid wastes, i.e., ISs, for a potential application in the field of industrial wastewater purification.

## 6. Prospect

In the current study, the photocatalyst removal efficiency of supported TiO_2_ has been close to 100%. Therefore, in a subsequent study, we will continue to optimise the performance of ISs/TiO_2_ photocatalyst by adjusting the process parameters. When the technology proposed in this paper is used for large-scale wastewater degradation, there may be two problems: the first is the recovery of the catalyst. When enough pollutants are absorbed, the recovery and recycling of the catalyst are worthy of further study. In addition, whether the catalyst can serve stably in a complex water environment needs to be considered. In addition, studies have shown that the removal efficiency of pollutants can be improved to some extent by the Fe-fenton reaction [68,69,70]. We will continue to conduct similar studies.

## Figures and Tables

**Table 1:** Chemical composition (wt.%) and particle size of the iron scales used in this study.

Composition	Fe	C	S	P_2_O_5_	SiO_2_	MnO	Al_2_O_3_	CaO	MgO	Diameter (μm)
Weight	73.85	0.016	0.009	0.051	0.058	0.347	0.008	0.131	0.017	5–100

**Table 2:** Absolute electronegativity, bandgap energy, calculated conduction band edge position and valence band edge position at the point of zero charge of the Fe_2_O_3_ particles and TiO_2_ particles.

Sample	Absolute Electronegativity (eV)	Bandgap Energy (eV)	Calculated Conduction Band Position (eV)	Calculated Valence Band Position (eV)
Fe_2_O_3_	5.88	2.10	0.33	2.43
TiO_2_	5.81	3.10	−0.24	2.86

## Data Availability

The raw data files can be downloaded and viewed at the following website: https://figshare.com/s/3a6d284803b5563c1585 (accessed on 11 March 2024).

## Supplementary Materials

The following supporting information can be downloaded at https://www.mdpi.com/article/10.3390/ma17184546/s1, Figure S1: Refinement of the X-ray diffraction patterns of the unloaded TiO_2_ particles and Fe_2_O_3_/TiO_2_ photocatalytic composites; Table S1: Detailed data on the crystal lattice constants of the unloaded TiO_2_ particles and Fe_2_O_3_/TiO_2_ photocatalytic composites.
