# Supplementary material for: Synthesis of Fe2O3/TiO2 Photocatalytic Composites for Methylene Blue Degradation as a Novel Strategy for High-Value Utilisation of Iron Scales
Source: Materials (Basel). 2024 Sep 16;17(18):4546. doi: 10.3390/ma17184546 (PMC11432992; doi:10.3390/ma17184546)
Supplement: Supplementary file 1 [file materials-17-04546-s001.zip › materials-3155398-supplementary.pdf]

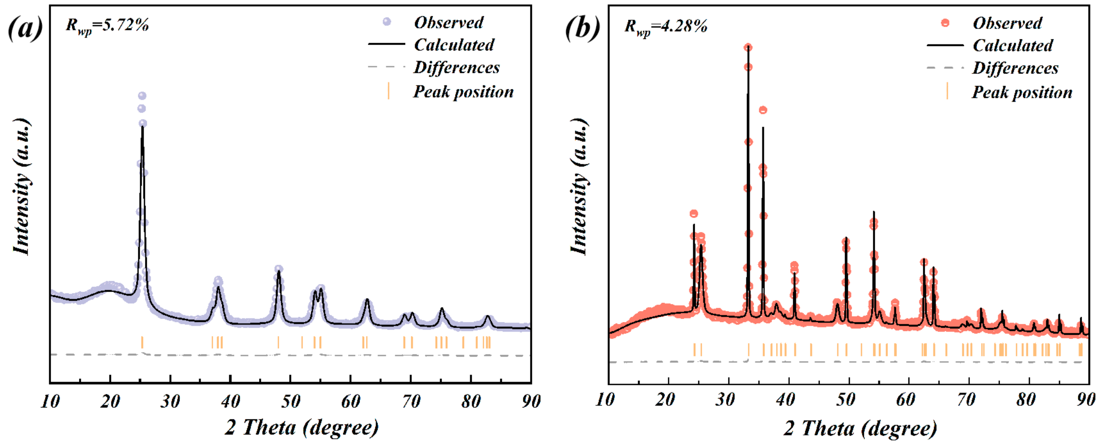

Figure S1. Refinement of the X-ray diffraction patterns of the unloaded TiO_2 particles and Fe_2O_3/TiO_2 photocatalytic composites.

Table S1. Detailed data on the crystal lattice constants of the unloaded TiO_2 particles and Fe_2O_3/TiO_2 photocatalytic composites.

	**Refinement Parameters**	**Phase**	**Cell Parameters**			**Bond Angle**		
	Rwp		a	b	c	α	β	γ
Fe_2O_3/TiO_2 photocatalytic composites	4.28%	Fe_2O_3	5.034	5.034	13.747	90.00	90.00	120.00
		TiO_2	3.788	3.788	9.500	90.00	90.00	90.00
unloaded TiO_2 particles	5.72%	TiO_2	3.790	3.790	9.491	90.00	90.00	90.00
